# *Jacobyana* Maulik, an Oriental flea beetle genus new for the Afrotropical Region with description of three new species from Central and Southern Africa (Coleoptera, Chrysomelidae, Alticinae)

**DOI:** 10.3897/zookeys.86.804

**Published:** 2011-03-19

**Authors:** Maurizio Biondi, Paola D’Alessandro

**Affiliations:** Dipartimento di Scienze Ambientali, University of L’Aquila, 67100 Coppito - L’Aquila, Italy

**Keywords:** Afrotropical Region, Oriental Region, Chrysomelidae, Alticinae, *Jacobyana*, new species

## Abstract

An Orientalflea beetle genus *Jacobyana* Maulik, 1926 including 7 species from India, Nepal, Vietnam and Sri Lanka, is reported in the Afrotropical Region for the first time. It is represented there by three new species, *Jacobyana bezdeki* **sp. n.**, *Jacobyana centrafricana* **sp. n.**, and *Jacobyana sudafricana* **sp. n.** Micrographs of male and female genitalia, scanning electron micrographs of some diagnostic morphological characters, a key to identification and distributional data for the new species, are provided.

## Introduction

The flea beetle genus *Jacobyana* was described by Maulik (1926) with the type species *Sphaerophysa piceicollis* Jacoby, 1889 from Burma (Myanmar). Subsequently, (Chen (1934, 1935) ascribed to this genus two taxa, *Jacobyana piceicollis* (Jacoby, 1889) var. *nigra* Chen, 1934 from Tonkin (Vietnam) and *Jacobyana nigrofasciata* Chen, 1935 from Sikkim (India). Later Scherer (1969) described *Jacobyana naini* from Uttar Pradesh (India). More recently *Jacobyana* was found in Panchthar, Nepal (*Jacobyana nepalica* Medvedev, 1990) and Sri Lanka (*Jacobyana ovata* Medvedev, 2001). Finally, Sprecher-Uebersax (2002) described two more species: *Jacobyana flurinae* and *Jacobyana serainae* from northern-eastern India.

In this paper we report the first records of *Jacobyana* for Sub-Saharan Africa and describe three new species: *Jacobyana bezdeki* sp. n., from Malawi, *Jacobyana centrafricana* sp. n., from Democratic Republic of Congo, and *Jacobyana sudafricana* sp. n., from Republic of South Africa. This extends considerably the geographical distribution of *Jacobyana* and widens already important Oriental component in the Afrotropical flea beetle fauna (Biondi and D’Alessandro 2010).

The Afrotropical Region, in fact, shares the highest number of flea beetle genera with the Oriental Region (26 of 102 genera in total) (Biondi and D’Alessandro 2010). The presence of some Oriental genera in the Afrotropical Region may be due to a possible Gondwanian origin: *Sanckia* Duvivier, 1891, which mainly occurs in Madagascar although species are found in the Sub-Saharan Africa and southern part of the Oriental Region; *Torodera* Weise, 1902, occurs in Sub-Saharan Africa and the Oriental Region, but it is absent from Madagascar; *Amphimela* Chapuis, 1875, *Nisotra* Baly, 1864, and *Paradibolia* Baly, 1875, occur in the Afrotropical, Oriental and Australian Regions; and *Bikasha* Maulik, 1931, occurs both on the Seychelles Islands and peninsula of Vietnam.

## Materials and methods

Material consisted of dried insects preserved in the institutions listed below. Further faunistic data on the *Jacobyana* species in Sub-Saharan Africa were collected during zoological collecting trips that were part of an Italian research project (PRIN 2004057217) aimed at interpreting the disjunct distribution of different plant and animal groups in the Mediterranean-South African regions. Specimens were examined and dissected using WILD MZ12.5 and LEICA M205C binocular microscopes. Photomicrographs were taken using a Leica DFC500 camera and the Auto-Montage Pro 2006 software (license number: 15224*syn2459*153a2112_maurizio_266836). Scanning electron micrographs were taken using a HITACHI TM-1000. Geographical coordinates of the localities are reported in degrees and minutes (DMD-WGS84 format); those included in square brackets were added by the authors.

Abbreviations. Morphology. LAED: length of median lobe of aedeagus; LAN: length of antennae; LB: total length of body; LE: length of elytra; LP: length of pronotum; LSP: length of spermatheca; WE: width of elytra; WP: width of pronotum.

Collections and depositories. BAQ: collection of M. Biondi preserved in the Dipartimento di Scienze Ambientali, University of L’Aquila, Italy; BMNH: The Natural History Museum, London, United Kingdom; SANC: South African National Collection, Plant Protection Research Institute, Pretoria, Gauteng, Republic of South Africa; TMSA: Transvaal Museum, Pretoria, Gauteng, Republic of South Africa.

## Taxonomy

### Genus Jacobyana

Maulik

Jacobyana Maulik 1926: 284, 302–303.

#### Type species.

*Sphaerophysa piceicollis* Jacoby, 1889: 195, by original designation. (Type locality: Burma).

#### Morphological remarks.

Based on newly examined material, morphological characteristic of *Jacobyana* is revised and updated with respect to the original description (Maulik 1926). Body roundish, strongly convex (Figs 2, 8, 14). Head with evident setiferous punctures (Figs 3, 9, 15); antenna short, generally not reaching pronotal base (Figs 2, 8, 14); third antennomere clearly thinner than first two antennomeres and about as long or longer than fourth and fifth together (as in *Jacobyana piceicollis* and *Jacobyana flurinae*); distal antennomeres (7 through11) distinctly longer than middle ones (3 through 6). Pronotum (Figs 4, 10, 16) clearly transverse (WP/LP > 2.2), anteriorly narrower than posteriorly, without antebasal furrow; lateral margins distinctly bordered, with anterior setiferous pore rearward towards middle of pronotal side; posterior margin not bordered, clearly sinuous. Elytral punctation (Fig. 17) arranged in regular rows; interstriae flat. Hind femur strongly enlarged; all femora ventrally with a large and generally deep furrow as long as femoral length, with smooth surface, to receive tibiae in resting position; hind tibia dorsally clearly channeled with distinct apical spur; tarsal claw sub-appendiculate (Fig. 7). Ventral surface (Figs 5, 11, 18) with numerous setiferous punctures, generally rather uniformly distributed; procoxal cavities posteriorly open; metasternum about as long as first abdominal sternite; elytral epipleura wide and slightly concave.

Metafemoral spring (Fig. 19) similar to springs of *Psylliodes* morpho-group (Furth & Suzuki 1998) but likely constitutes a new morpho-group with dorsal lobe regularly curved, with rather long extended arm; basal edge straight, angled < 90° with central axis of dorsal lobe; dorsal edge of ventral lobe straight; basal angle of ventral lobe acute, short, apically pointed; recurve flange distinctly sclerotized.

#### Distribution.

**Figure 1.**
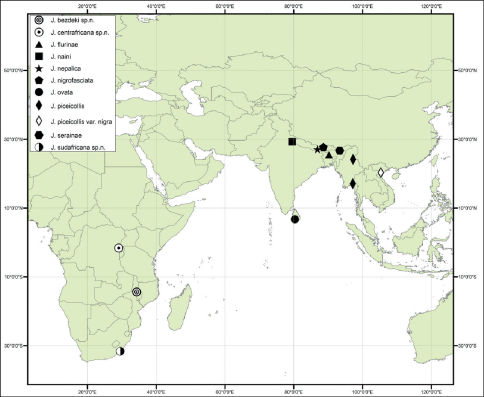
Geographical distribution of the genus *Jacobyana* Maulik.

Oriental (India, Nepal, Vietnam and Sri Lanka) (Medvedev 2009) and Afrotropical (Democratic Republic of Congo, Malawi, and Republic of South Africa) Regions (Fig. 1).

#### Notes.

*Jacobyana bezdeki* sp.n. (LB = 2.64 mm), *Jacobyana centrafricana* sp. n. (LB = 2.66 mm), and *Jacobyana sudafricana* sp.n. (LB ♂ = 2.25 mm and ♀ > 2.43 mm) are the smallest species of the genus (Sprecher-Uebersax 2002). All new Afrotropical species share a ratio of the length between antennomeres 3 and 4 (= 2) with *Jacobyana ovata* Medvedev from Sri Lanka (known only by a single female). However, all three African species are distinguishable from *Jacobyana ovata* by their smaller size (*Jacobyana ovata*: LB = 3.40 mm), interantennal space distinctly narrower than length of first antennomere (as broad as first antennomere in *Jacobyana ovata*) and, with the exception of some specimens of *Jacobyana sudafricana* sp. n., by the colour of the dorsal integuments, never uniformly black (entirely black in *Jacobyana ovata*).

### Jacobyana bezdeki sp. n.

urn:lsid:zoobank.org:act:8429491E-26FC-469C-B106-8B97C94A3D4B

#### Type series.

Holotype ♂: MALAWI: Dedza env. [~ 14°23'S, 34°19'E], 06–13.i.2002, J. Bezděk leg. (BAQ).

#### Diagnosis.

*Jacobyana bezdeki* sp. n. is different from *Jacobyana centrafricana* sp.n. and *Jacobyana sudafricana* sp.n. in having dorsal integuments reddish-brown (integuments are entirely or almost entirely black in *Jacobyana sudafricana* sp. n. and black with reddish elytral apex in *Jacobyana centrafricana* sp.n.). Other distinctive characters are: head with frons and vertex distinctly raised (Figs 3, 9, 15); punctation medially absent in distal part of first abdominal sternite and in last abdominal sternite (Figs 5, 11, 18); median lobe of the aedeagus in ventral view laterally sub-parallel and apically widely rounded (Figs 6, 13, 20).

#### Description.

##### Holotype

**Figures 2–7.**
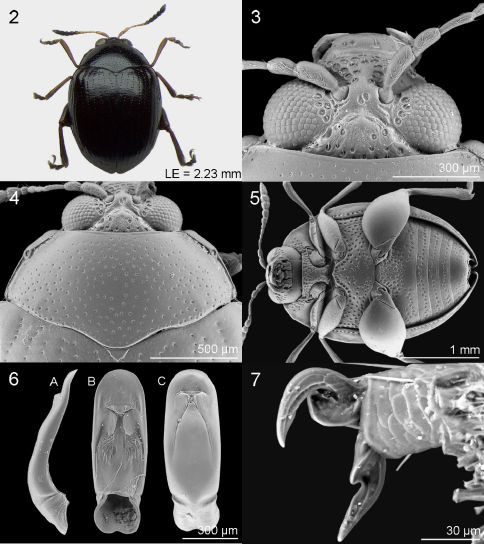
*Jacobyana bezdeki* Biondi & D’Alessandro,sp. n. ♂, holotype (Malawi). **2** habitus **3** head **4** pronotum **5** ventral parts **6** median lobe of aedeagus, in lateral (**6A**), ventral (**6B**), dorsal (**6C**) view **7** tarsal claws.

♂. Dorsal integument (Fig. 2) reddish-brown with evident metallic reflection; head, pronotum and elytral punctation darkened. Body elliptical (LB = 2.64 mm), weakly elongate, strongly convex. Maximum pronotal width at base (WP = 1.38 mm); maximum elytral width at basal third (WE = 1.80 mm).

##### Frons and vertex

(Fig. 3) distinctly raised, with clearly wrinkled and punctulate surface and distinct setiferous punctures; frontal tubercles indistinguishable; interantennal space distinctly smaller than length of first antennomere, medially with two jointed setiferous pores clearly impressed on sub-smooth and punctulate surface; frontal carina not raised; clypeus triangular with large setiferous punctures; labrum sub-rectangular, reddish; palpus yellowish; eye sub-elliptical, normally sized; antenna much shorter than body length (LAN = 0.94 mm; LAN/LB = 0.36), with antennomeres 1–6 entirely pale, antennomere 7 partially darkened, antennomeres 8–11 clearly darkened; antennomeres 1–2 and 7–11 clearly enlarged; length of each antennomere proportional to numerical sequence 22:13:18:9:9:8:12:11:12:12:23 (right antenna).

##### Pronotum

(Fig. 4) sub-trapezoidal, strongly transverse (LP = 0.66 mm; WP/LP =2.08), laterally clearly and evenly rounded, basally as wide as elytra; basal margin distinctly sinuous, not bordered; lateral margin distinctly bordered, with anterior setiferous pore rearward little before middle of pronotal side; punctures densely and uniformly distributed on very finely and sparsely punctulate surface; punctures small but clearly impressed. Scutellum very small, sub-triangular, with sub-smooth surface.

##### Elytra

moderately elongate (LE = 2.23 mm; LE/LP = 3.36), covering entire pygidium, laterally clearly arcuate, apically jointly rounded; punctures small but clearly impressed, arranged in 9 regular rows (+ 1 short scutellar row); surface sub-smooth with dense punctulation; interstriae flat; humeral callus very weakly prominent; macropterous metathoracic wings.

##### Legs

with partially darkened femur and reddish tibia and tarsi; hind tibia straight without dentate external margin; apical spur of hind tibiae short, reddish. First anterior and middle tarsomeres slightly dilated with adhesive setae on ventral side (Fig. 12).

##### Ventral parts

(Fig. 5) dark-brown, with dense and rather uniformly distributed setiferous punctures, but medially sparser or absent on prosternum, metasternum, distal part of first abdominal sternite and last abdominal sternite; last abdominal sternite without special preapical impressions.

##### Median lobe of aedeagus

(Fig. 6) short and robust (LAED = 0.91 mm; LE/LAED = 2.44), in ventral view laterally sub-parallel, apically widely rounded; ventral sulcus very wide, clearly impressed, with partially wrinkled surface and two paired short longitudinal carinae and numerous small protruding structures; dorsal sulcus poorly-developed; dorsal ligula well-developed, apically acute; median lobe in lateral view clearly arcuate at basal third and slightly sinuous in apical part; surface of median lobe with pores, especially on ventral side of apical part.

#### Etymology.

This species is named after its collector J. Bezděk (Czech Republic), valued specialist of Chrysomelidae.

#### Distribution.

Malawi (Fig. 1). Eastern Afrotropical chorotype (EAF) (Biondi and D’Alessandro 2006).

#### Ecological notes.

Host plant is unknown.

### Jacobyana centrafricana sp.n.

urn:lsid:zoobank.org:act:55ECC82E-16CE-4D8D-BABD-EE02D92840BF

#### Type series.

Holotype ♂: ZAIRE [= REPUBLIC OF CONGO]: Nord Kivu, Lac Mugunga [~ 1°37'S, 29°32'E], 1520 m, 16.vii.1990, G. Carpaneto & S. Zoia leg. (BAQ).

#### Diagnosis.

*Jacobyana centrafricana* can be distinguished from *Jacobyana bezdeki* sp. n. and *Jacobyana sudafricana* sp. n. by the following features: the dorsal integuments being black with elytral apex clearly reddish and legs distinctly paler (reddish-brown in *Jacobyana bezdeki* sp. n.; more diffusely black in *Jacobyana sudafricana* sp. n.); the basal margin of the pronotum is slightly more sinuous (Figs 4, 10, 16); punctures of the ventral side of the body more densely impressed (Figs 5, 11, 18); the median lobe of the aedeagus in ventral view is clearly lanceolate, laterally with the maximum width about middle and with acutely rounded apex (Figs 6, 13, 20).

#### Description.

##### Holotype

**Figures 8–13.**
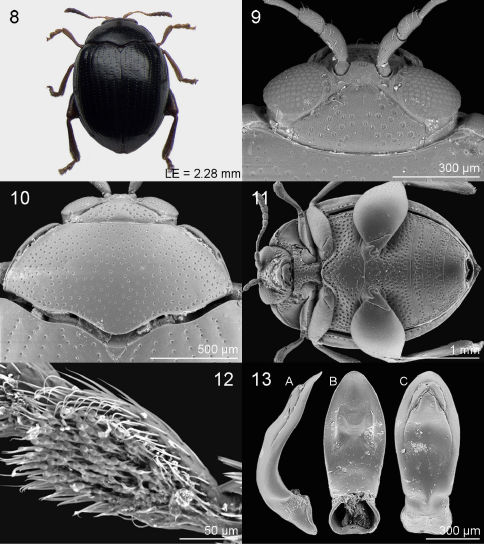
*Jacobyana centrafricana* Biondi & D’Alessandro, sp. n. ♂, holotype (Democratic Republic of Congo). **8** habitus **9** head **10** pronotum **11** ventral parts **12** ventral side of first anterior tarsomere in male **13** median lobe of aedeagus, in lateral (**13A**), ventral (**13B**), dorsal (**13C**) views.

♂. Dorsal integument (Fig. 8) entirely black with evident metallic reflection; elytral apex clearly reddish. Body roundish (LB = 2.66 mm), strongly convex. Maximum pronotal width at base (WP = 1.39 mm); maximum elytral width at basal third (WE = 1.88 mm).

##### Frons and vertex

(Fig. 9) with clearly shagreened and punctate surface, and distinct setiferous punctures; frontal tubercles subtriangular, blackish, very scarcely delimited, with shagreened surface; frontal grooves distally deep, particularly along ocular margin; interantennal space distinctly narrower than length of first antennomere, medially with two setiferous pores; frontal carina not raised; clypeus triangular with large setiferous punctures; labrum sub-rectangular, distally brownish; palpus yellowish; eye sub-elliptical, normally sized; antenna much shorter than body length (LAN = 1.00 mm; LAN/LB = 0.38), entirely pale, but with antennomeres 8–11 clearly blackened; antennomeres 1–2 and 7–11 clearly enlarged; length of each antennomere proportional to numerical sequence 23:14:20:10:9:8:14:14:16:16:20 (right antenna).

##### Pronotum

(Fig. 10) sub-trapezoidal, strongly transverse (LP = 0.68 mm; WP/LP = 2.06), laterally clearly and evenly rounded, basally as wide as elytra; basal margin distinctly sinuous, not bordered; lateral margin distinctly bordered, with anterior setiferous pore rearward at middle of pronotal side; punctures densely and uniformly distributed on shagreened and very finely punctulate surface; punctures small but clearly impressed. Scutellum very small, half-roundish, with sub-smooth surface, medially clearly depressed.

##### Elytra

moderately elongate (LE = 2.28 mm; LE/LP = 3.37), covering entire pygidium, laterally strongly arcuate, apically jointly rounded; punctures small but clearly impressed, arranged in 9 regular rows (+ 1 short scutellar row); surface sub-smooth with very finely and sparsely punctulation; interstriae flat; humeral callus very weakly prominent; macropterous metathoracic wings.

##### Legs

with blackish femur and tibia but with paler tarsi; hind tibia straight with no dentate external margin; apical spur of hind tibia short, reddish. First anterior and middle tarsomeres slightly dilated with adhesive setae on ventral side (Fig. 12).

##### Ventral side

(Fig. 11) blackish, with very dense and rather uniformly distributed setiferous punctures, sparser or absent in middle part of prosternum, metasternum and last abdominal sternite; last abdominal sternite without special preapical impressions.

##### Median lobe of aedeagus

(Fig. 13) short and robust (LAED = 0.93 mm; LE/LAED = 2.46), in ventral view clearly lanceolate, laterally with maximum width about at middle; apex acutely rounded; ventral sulcus very wide, moderately impressed, without any evident carinae or sulci but medially weakly protruding; dorsal sulcus obliterate; dorsal ligula well developed, apically acutely rounded; median lobe in lateral view basally strongly arcuate at basal third and slightly sinuous in distal half; apex slightly bent in ventral direction.

#### Etymology.

This species name refers to the geographic region where it lives and means “from Central Africa”.

#### Distribution.

Republic of Congo (Nord Kivu) (Fig. 1). Central Afrotropical chorotype (CAT) (Biondi and D’Alessandro 2006).

#### Ecological notes.

Host plant is unknown.

### Jacobyana sudafricana sp. n.

urn:lsid:zoobank.org:act:2BFA5865-4DA2-41DC-A189-A444C992F2AD

#### Type series.

Holotype ♂: REPUBLIC OF SOUTH AFRICA: Eastern Cape Province, Port St. Johns, Silaka Nature Reserve, 31°39.45'S, 29°30.14'E, 10.xi.2006, G. Osella leg. (BAQ). Paratypes: same locality, date and collector as the holotype, 2 ♀♀ (BAQ; SANC); ditto, E. Colonnelli leg., 2 ♀♀ (BAQ; TMSA); South Africa, Pondoland, Port St. Johns [31°38'S, 29°32'E], 29.i-5.ii.1924, R.E. Turner leg., 2 ♀♀ (BMNH).

#### Diagnosis.

**Figures 14–21.**
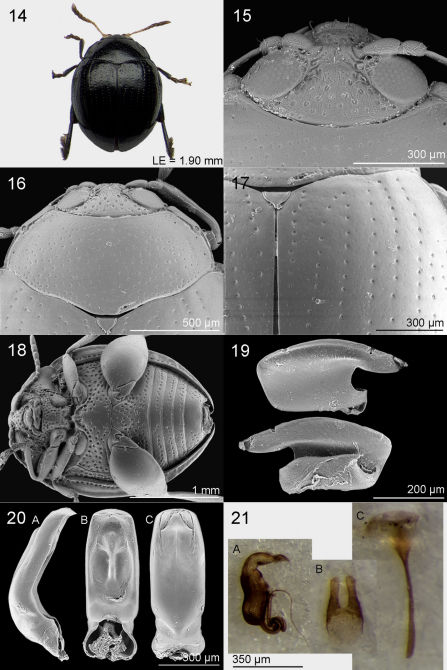
*Jacobyana sudafricana* Biondi & D’Alessandro, sp. n. ♂, holotype (Republic of South Africa). **14** habitus **15** head **16** pronotum **17** elytral surface **18** ventral parts **20** median lobe of aedeagus, in lateral (**20A**), ventral (**20B**), dorsal (**20C**) views. *Jacobyana sudafricana* Biondi & D’Alessandro, sp. n. ♀, paratype (Republic of South Africa). **19** metafemoral spring **21A** spermatheca **21B** vaginal palpi **21C** tignum.

*Jacobyana sudafricana* sp. n. is the smallest speciesof the genus (LB ♂ = 2.25 mm); it is also distinctly smaller than *Jacobyana bezdeki* sp. n. (LB = 2.64 mm) and *Jacobyana centrafricana* sp.n. (LB = 2.66 mm). The following characters separate *Jacobyana sudafricana* from the rest African species: pronotal surface laterally slightly depressed (Figs 4, 10, 16); elytra laterally more rounded (Figs 2, 8, 14); punctation of the ventral part medially absent on the last four abdominal sternites (Figs 5, 11, 18); median lobe of aedeagus in ventral view laterally sub-parallel with widely sub-truncate apex (Figs 6, 13, 20).

#### Description.

##### Holotype

♂. Dorsal integument (Fig. 14) entirely black with evident metallic reflection; tip of elytral apex reddish. Body roundish (LB = 2.25 mm), strongly convex. Maximum pronotal width at base (WP = 1.28 mm); maximum elytral width at basal fourth (WE = 1.71 mm).

##### Frons and vertex

(Fig. 15) with clearly shagreened and finely punctulate surface, with distinct setiferous punctures; frontal tubercles subtriangular, brownish, scarcely delimited, with shagreened surface; frontal grooves distally deep, particularly along ocular margin; interantennal space distinctly narrower than length of first antennomere, medially with two setiferous pores not well delimited; frontal carina not raised; clypeus triangular with large setiferous punctures; labrum sub-rectangular, distally brownish; palpus yellowish; eye sub-elliptical, normally sized; antenna much shorter than body length (LAN = 1.00 mm; LAN/LB = 0.44), entirely pale but with antennomeres 5–11 very slightly obfuscate; antennomeres 1–2 and 7–11 clearly enlarged; length of each antennomere proportional to numerical sequence 18:14:22:11:10:8:12:14:16:16:22 (right antenna).

##### Pronotum

(Fig. 16) sub-trapezoidal, strongly transverse (LP = 0.55 mm; WP/LP = 2.32), laterally clearly rounded, basally as wide as elytra; pronotal surface laterally weakly depressed; basal margin not bordered, sinuous; lateral margin distinctly bordered, with anterior setiferous pore rearward at middle of pronotal side; punctures small but distinctly impressed, few densely but uniformly distributed on very finely and sparsely punctulate surface. Scutellum small, sub-triangular, apically with very small median tooth; surface smooth, anteriorly finely rugose.

##### Elytra

moderately elongate (LE = 1.90 mm; LE/LP = 3.37), covering entire pygidium, laterally strongly arcuate, apically jointly rounded; punctures small and moderately impressed (Fig. 17), arranged in 9 regular rows (+ 1 short scutellar row); surface very finely and sparsely punctulate; interstriae flat; humeral callus very weakly prominent; macropterous metathoracic wings.

##### Leg

entirely reddish-brown with partially blackened femur; hind tibia very slightly curved with no dentate external margin; apical spur of hind tibia short, reddish. First anterior and middle tarsomeres very weakly dilated, with adhesive setae on ventral side (Fig. 12).

##### Ventral surface

(Fig. 18) blackish, with dense and rather uniformly distributed setiferous punctures, medially sparser or absent on prosternum, metasternum and last four visible abdominal sternites; last abdominal sternite without special preapical impressions.

##### Median lobe of aedeagus

(Fig. 20) short and robust (LAED = 0.75 mm; LE/LAED = 2.45), in ventral view laterally sub-parallel in basal 2/3 and slightly convergent in apical third; apex widely sub-truncate; ventral sulcus very wide, clearly impressed, with evident longitudinal median carina distally clearly expanded, with numerous small protruding structures; dorsal sulcus obliterate; dorsal ligula well-developed, apically sub-triangular; median lobe in lateral view very strongly curved at basal third and clearly bent in ventral direction at apex; surface of median lobe with pores, especially on ventral side of apical part.

#### Variation.

♂ (n = 1): LE = 1.90 mm; WE = 1.71 mm; LP = 0.54 mm; WP = 1.28 mm; LAN = 1.00 mm; LAED = 0.75 mm; LB = 2.25 mm; LE/LP = 3.45; WE/WP = 1.34; WP/LP = 2.32; LE/LAED = 2.53; LAN/LB = 0.44. ♀ (n = 6; mean and standard deviation): LE = 2.25 ± 0.10 mm; WE = 2.01 ± 0.08 mm; LP = 0.65 ± 0.02 mm; WP = 1.46 ± 0.06 mm; LAN = 1.10 ± 0.03 mm; LSP = 0.45 ± 0.02 mm; LB = 2.61 ± 0.10 mm; LE/LP = 3.46 ± 0.08; WE/WP = 1.38 ± 0.02; WP/LP = 2.25 ± 0.08; LE/LSP = 4.96 ± 0.27; LAN/LB = 0.42 ± 0.01.

Paratypes (all females) very similar in shape, sculpture and color to the holotype, but distinctly bigger. Tip of elytral apex variable in color from entirely black to partially reddish. Spermatheca (Fig. 21A) with sub-reniform and elongate basal part, clearly more developed than apical part; apical part with distinct collum and apex; appendix evident; ductus elongate, sub-apically inserted, with several coils in proximal part. Vaginal palpi and tignum as in Fig. 21B, 21C.

#### Etymology.

This species name refers to the geographic region where it lives and means “from Southern Africa”.

#### Distribution.

Republic of South Africa (Eastern Cape Province) (Fig. 1). Southern-Eastern African chorotype (SEA) (Biondi and D’Alessandro 2006).

#### Ecological notes.

Host plant is unknown. Biome: Forest (Rutherford and Westfall 1994). Veld type: Coastal Forest and Thornveld (Acocks 1988).

## Key to species

**Table:** 

1	Dorsal integuments reddish-brown. Frons and vertex distinctly raised (Fig. 3). Median lobe of aedeagus (Fig. 6) short and robust, in ventral view laterally sub-parallel, apically widely rounded; ventral sulcus very wide, clearly impressed; in lateral view basally clearly arcuate at basal third and slightly sinuous in apical part	*Jacobyana bezdeki* sp. n.
–	Dorsal integuments entirely black or with reddish elytral apex. Frons and vertex flat (Figs 9, 15)	2
2	Dorsal integuments black with clearly reddish elytral apex. Abdominal sternites with uniformly and densely impressed punctures (Fig. 11). Median lobe of aedeagus (Fig. 13) in ventral view clearly lanceolate with acutely rounded apex; in lateral view strongly arcuate at basal third and slightly sinuous in distal half with apex slightly bent ventrally	*Jacobyana centrafricana* sp. n.
−	Dorsal integuments entirely black (sometimes only with slightly reddish tip of elytra). Distal abdominal sternites medially without punctation (Fig. 18). Median lobe of aedeagus (Fig. 20) in ventral view apically widely sub-truncate, with ventral sulcus very wide with evident longitudinal median carina; median lobe in lateral view very strongly curved at basal third with apex clearly bent ventrally	*Jacobyana sudafricana* sp. n.

## Supplementary Material

XML Treatment for Jacobyana

XML Treatment for Jacobyana bezdeki

XML Treatment for Jacobyana centrafricana

XML Treatment for Jacobyana sudafricana
